# Cone-beam CT evaluation of root canal morphology of maxillary and mandibular premolars in a Turkish Cypriot population

**DOI:** 10.1038/bdjopen.2015.6

**Published:** 2016-01-29

**Authors:** Berkan Celikten, Kaan Orhan, Umut Aksoy, Pelin Tufenkci, Atakan Kalender, Fatma Basmaci, Pervin Dabaj

**Affiliations:** 1 Ankara University, Faculty of Dentistry, Department of Endodontics, Ankara, Turkey; 2 Ankara University, Faculty of Dentistry, Department of Dentomaxillofacial Radiology, Ankara, Turkey; 3 Near East University, Faculty of Dentistry, Department of Endodontics, Nicosia, Cyprus; 4 Department of Endodontics, Faculty of Dentistry, Mustafa Kemal University, Hatay, Turkey

## Abstract

**Objectives:**

Because of economic and political issues, Turkish Cypriots have been emigrating from Cyprus since the 1920s, especially to the United Kingdom, other European countries and Australia. Recently, according to the UK House of Commons, Home Affairs Committee, ~300,000 Cypriot Turks were living in the United Kingdom. However, this ethnic population residing in the United Kingdom has been insufficiently analysed. Although many Turkish Cypriots have been living abroad, little is known about the dental characteristics of this group. Premolar teeth, especially maxillary premolars, pose great challenges in endodontic treatment because of the number of roots and canals, and the variation in the configurations of the pulp cavity. Thus, it was considered valuable to determine the morphological characteristic of premolar teeth in a Turkish Cypriot population to aid clinicians in performing endodontic treatment in this ethnic population.

**Materials and Methods:**

The sample for this cross-sectional study consisted of a retrospective evaluation of cone-beam CT scans of 263 adult patients (age range 16–80 years). The number of roots and their morphology, the number of canals per root and the canal configuration were examined. The root canal configurations were also classified according to the scheme of Vertucci in the maxillary and mandibular premolar teeth. Pearson’s *χ*^2^-test was performed among canal configurations, sides and gender (*P*⩽0.05).

**Results:**

In the present study, most root canal configurations were type IV (76.8%) and type I (49.4%) in the maxillary first and second premolars, respectively, whereas most root canal configurations were type I (93%) in both mandibular first and second premolars. In total, four (0.9%) teeth in the maxillary first premolars and two (0.4%) teeth in the maxillary second molar premolars had three roots.

**Conclusions:**

This is the first population-based study to focus solely on Turkish Cypriots’ root canal anatomy. Our findings will be valuable for dental professionals who treat many Turkish Cypriot patients, in the United Kingdom, Australia and other countries.

## Introduction

Successful endodontic treatment depends on shaping, disinfecting and filling the root canal system. Teeth with anatomical variation are an important issue in root canal treatment. Missing root canals may contain necrotic tissue and microorganisms. Over time, the microorganisms can proliferate and cause apical periodontitis. Thus, clinicians should be aware of complex root canal structures.^1^

Premolar teeth root canal treatment is a challenge for clinicians. Generally, first maxillary premolars have two roots and two canals (56%) and first maxillary premolars have one root with two canals (40%). However, several studies have shown that maxillary and mandibular premolars teeth often have additional roots and canals.^2–6^ Vertucci and Gegauff^5^ stated that three root canals were observed in 5–6% of maxillary premolars, whereas Caliskan *et al.*^7^ found no three-separate-rooted first maxillary premolars in their study. Typically, second maxillary premolars have one root with one oval-shaped canal. However, Ok *et al.*^8^ stated that one-canal second maxillary premolars were observed in 59.7% of cases, two canals in 40% and three canals in 0.30% in their study. Consistent with this, Vertucci *et al.*^9^ reported an incidence of 1% three-rooted-plus-three-canal second maxillary premolars. Mandibular premolars usually have one root with a canal (54–88.5%).^10^ However, mandibular premolars can also show various root canal configurations, such as maxillary premolars. Multiple canals have been reported in mandibular premolars, ranging from 11.5 to 46% of teeth.^11,12^

Various methods have been used to evaluate root canal morphology in previous studies.^7,9,13^ Generally, these methods involved polyester resin impressions, producing transparent samples and taking radiographs in the mesiodistal and/or buccolingual directions. However, recently cone-beam computed tomography (CBCT) has been used to evaluate root canal anatomy because it facilitates diagnosis and provides clinicians with three-dimensional information about the morphology of roots and their divergence.^14–17^

For economic and political reasons, many Turkish Cypriots have emigrated from Cyprus since the 1920s, especially to the United Kingdom, other European countries and Australia. Recently, according to the UK House of Commons Home Affairs Committee,^18^ there are ~500,000 people of Turkish origin in the United Kingdom; ~150,000 Turkish nationals and ~300,000 Cypriot Turks. Unfortunately, analyses of ethnic populations residing in various countries have been insufficient in terms of dental characteristics. Although many Turkish Cypriots now reside abroad, little is known about their root canal configurations. Such knowledge would facilitate endodontic treatment in this population, especially in premolar teeth, which pose great challenges for endodontic treatment because of the numbers of roots, canals and the variation in the configurations of the pulp cavity. Thus, it was considered valuable to determine the root canal configurations of premolar teeth in a Turkish Cypriot population using CBCT to aid clinicians in performing endodontic treatment in this ethnic population.

## Materials and methods

The sample for this cross-sectional study consisted of 263 patients (age range, 16–80 years) seeking routine dental care at the University Dental Hospital. All of the proposed subjects agreed to participate in this study, and all gave written inform consent. The study was approved by the ethics committee of the university.

Digitised CBCT images of mandibular premolars were collected from patients who had undergone CBCT scanning for diagnostic purposes. Premolars with immature apices, apical periodontitis, root canal fillings, and post and crown restorations were excluded. Cases where the anatomy was compromised by physiological or pathological processes and unclear root canal morphology were also excluded. In total, 882 maxillary and 954 mandibular premolar teeth were evaluated in terms of root canal configuration.

CBCT scans (Newton 3G, Quantitative Radiology s.r.l., Verona, Italy) used a 9-inch field of view to include the mandibular anatomy. All CBCT exposure was perform with the minimum exposure necessary for adequate image quality by an experienced licensed radiologist. The as low as reasonable achievable principle was followed. Axial, coronal and cross-section images were used to evaluate root canal anatomy. All of the constructions and measurements were performed on a 21.3-inch flat-panel colour-active matrix thin-film-transistor (TFT) medical display (NEC MultiSync MD215MG, Munich, Germany) with a resolution of 2,048×2,560 at 75 Hz and 0.17-mm dot pitch, operated at 11.9 bits. All of the CBCT images were evaluated retrospectively by two endodontists and one oral and maxillofacial radiologist with at least 10 years’ experience using CBCT device software (NNT 4.6, QR, Verona, Italy). An interexaminer calibration based on the anatomic diagnosis of CBCT images had been previously performed to assess data reliability. CBCT images were evaluated and the following were observed: (i) the number of roots and canals; (ii) the canal configuration in each root using Vertucci’s classification (2005); and (iii) the frequency of additional roots.

The observers evaluated the images twice with a 1-week interval between assessments. Intra- and inter-examiner reliability were determined. Wilcoxon’s matched-pairs signed rank test was used for intraobserver, whereas interobserver reliability was assessed by the intraclass correlation coefficient (ICC) and the coefficient of variation. Values for the ICC range from 0 to 1. ICC values higher than 0.75 show good reliability and the low coefficient of variation demonstrates the precision error, an indicator for reproducibility.^19^ Relationships among gender and sides with the incidence of additional canals were determined using the *χ*^2^-test. Differences were considered significant when *P*>0.05.

## Results

CBCT evaluations revealed no intraobserver variance for the observers (*P*>0.05). Overall measurement consistency for observer 1 was rated at 91.1%, and those for observers 2 and 3 were 89.3% and 90.2%, respectively. All of the measurements were highly correlated for the observers, and no significant difference was evident for repeated measurements by the observers (*P*>0.05). ICCs between the three observers ranged from 0.848 to 0.997. There was high interobserver agreement. The high ICC and low coefficient of variation demonstrated that the procedure was standardised between the evaluations and measurements performed by the observers. No significant difference was seen in any of the variables (*P*>0.05).

The results of the study are presented in Tables 1,2,3. Of the 437 maxillary first premolars, 4 (0.9%) teeth had three roots with three canals at the apex, 196 (44.8%) teeth had two roots with two canals and 236 (53.7%) had one root. The number and percentage of canals with a single root with two canals at the apex were 143 (32.6%) in maxillary first premolars. The frequency distribution of the number of root canals did not differ on the left and right sides (*P*>0.05). The maxillary first premolar group with single roots contained 62 (14%) females and 78 (17.7%) males with a Vertucci’s type IV root canal anatomy. In males and females, there were 18 (4%) and 53 (12%) with type II canal anatomy; the difference was statistically significant (*P*<0.05). In those with two roots with two canals at the apex, 196 (44.6%) showed type IV root canal anatomy. In four (0.9%) teeth, three roots with three canals at the apex were found in maxillary first premolars, all in males (Table 1).

**Figure 1:**
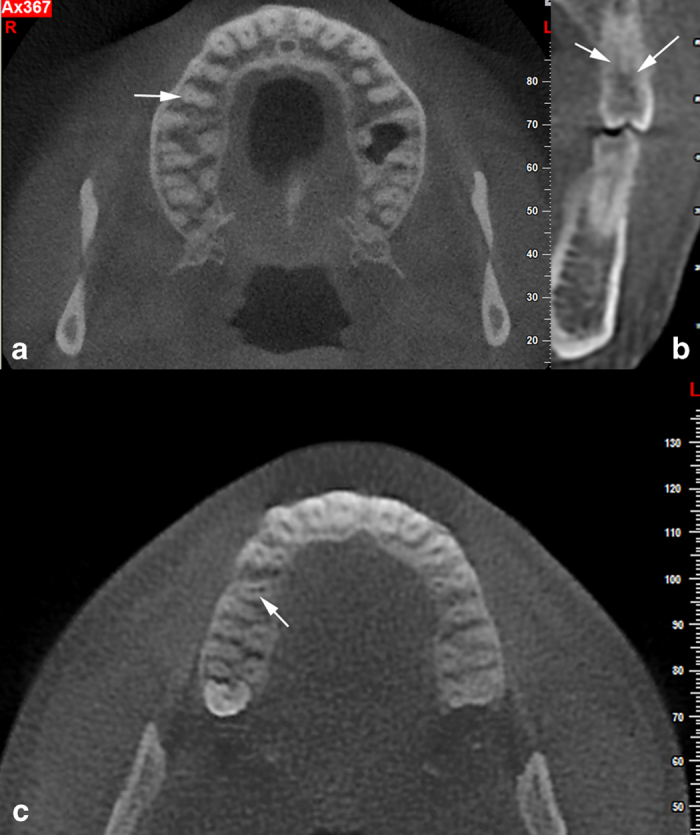
(**a**, **b**) Axial and cross-section CBCT images showing two rooted two canals maxillary second premolar. (**c**) Axial CBCT image showing type II maxillary second premolar (arrows).

In the second maxillary premolar group, single roots were seen in 115 (25.8%) females and 105 (23.5%) males with Vertucci’s type I root canal anatomy. There were 74 (16.5%) female and 50 (11.1%) male teeth with type II canal anatomy. Type IV canal anatomy was found in 21 (4.6%) female and 26 (5.8%) male teeth. Only two (0.4%) teeth exhibited three roots with three canals at the apex. In total, two roots with two canals at the apex were found with type IV canal anatomy in 24 (7.6%) teeth. No gender or side difference was found in the second premolars (*P*>0.05; Table 2). (Figure 1).

**Figure 2:**
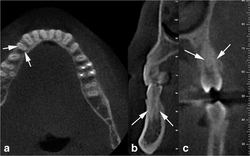
(**a**, **b**) Axial and cross-section CBCT image shows showing type V mandibular first premolar. (**c**) Cross-sectional image showing type II maxillary first premolar.

Of the 954 mandibular premolars, 886 (92.8%) teeth had a single root with one canal at the apex, followed by 36 (3.6%) teeth with two roots with two canals at the apex. The frequency distribution of the number of root canals did not differ by side (*P*>0.05). In the present study, most root canal configurations were type I (92.8%) and type V (3.7%) in mandibular first and second premolars, respectively (Figure 2). The least common root canal configurations were type IV (0.2%) and type II (0.7%) in mandibular first and second premolars, respectively (Table 3).

## Discussion

Success in endodontic treatment requires an understanding of canal anatomy and morphology. To achieve endodontic success, all of the canals must be debrided, disinfected, shaped and obturated completely.^20^ Reasons for failure of root canal treatment include an untreated canal, incomplete debridement and incomplete obturation.^7^ Thus, careful clinical and radiographical examinations are essential for successful endodontic treatment.

The present study provides a detailed investigation of the root and canal morphology of mandibular permanent molars in a Turkish Cypriot population using CBCT. Many techniques have been used to assess root canal morphology and configuration, such as macroscopic sections, transparent samples, polyester resin impressions and CBCT. Recently, CBCT has been used because it is considered an excellent method for the three-dimensional evaluation of root canal morphology.^21^

Previous studies have investigated root canal morphology and root numbers in premolars teeth. Bellizzi and Hartwell^22^ demonstrated that maxillary first premolar teeth had one canal in 6.2%, two canals in 90.5% and three canals in 3.3% of cases. Caliskan *et al.*^7^ reported that maxillary first premolar teeth had one canal in 3.92%, two canals in 96.7% and no case with three canals in a Turkish population. Likewise, in a Turkish population, Kartal *et al.*^6^ reported one canal in 8.66%, two canals in 89.64% and three canals in 1.66%. In studies on maxillary second premolar teeth, Pineda and Kuttler^23^ reported finding one canal in 55% and two canals in 45%. Vertucci *et al.*^9^ and Bellizzi and Hartwell^22^ in maxillary second premolar teeth reported one canal in 48 and 40.3%, two canals in 51 and 58.6% and three canals in 1 and 1.1%, respectively.

Another study of root canal configurations in first and second maxillary premolars found 60% type IV and 38% type I in males, and 63% type IV and 34% type IV in females in a Turkish population. A study of root canal morphology in maxillary and mandibular premolars in a Turkish population by Ok *et al.*^8^ reported root canal frequencies for the maxillary first premolar teeth of two canals (86.2%) and type IV (76.9%) configuration, and one canal (59.7%) and type I (54.5%) canal configuration for the second premolar. Liu *et al.*^24^ reported that mandibular premolars had a single canal in 65.2% and a double canal in 26.1% in a Chinese population. Likewise, in a study of a Japanese population using radiography, mandibular premolars had one canal (80.6%).^25^ In a Jordanian population and in an Iranian population, mandibular premolars had type I canals in 58.2% and 88.5%, respectively.^12,26^

In the present study, most root canal configurations were type IV (76.8%) and type I (49.4%) in maxillary first and second premolars, respectively. The least common canal root canal configurations were type V (0.6%) and type VI (0.2%) in maxillary first and second premolars, respectively. In a study by Ok *et al.*^8^, most root canal configurations were type IV (76.9%) and type I (54.5%) in the maxillary first and second premolars, respectively, in a Turkish population, similar to our results.

In this study, a single canal at the apex was seen in 21.1% and 78.9%, two canals in 77.4% and 20.3%, and three canals in 0.9% and 0.4% maxillary first and second premolars, respectively. In addition, maxillary first and second premolars with three canals at the apex were found in 6 (0.6%) teeth, all in males.

Particularly in Turkish root canal configuration studies, Caliskan *et al.*^7^ reported one canal at the apex in 9.8% and 72%, two canals in 90.1% and 28%, and three canals in 0% and 0% in maxillary first premolars and second premolars, respectively. According to Kartal *et al.*^6^, one canal at apex was seen in 9.66% and 54.9%, two canals in 88.6% and 44.3%, and three canals in 1.66% and 0.6% in maxillary first premolars and second premolars, respectively. Our results were largely similar. These slightly divergent results may be explained by methodological differences among the studies or variation in sample size, ethnic origin and regional background of the samples used.

The occurrence of a single canal in the mandibular first premolar was reported from 54 to 88.5%, whereas multiple canals were reported from 11.5 to 46%.^13–15^ Bolhari *et al.*^27^ reported mandibular premolars with a single canal at 91.24 and 8.75% with more than one canal. According to Vertucci’s classification, the type I configuration of the root canal system is more frequent (67.39%) than the other configurations. Vertucci^28^ reported type I in 70%, type II in 0%, type III in 4%, type IV in 1.5% and type V in 24%. According to Velmurugan and Sandhya^29^, Parekh *et al.*^30^ and Liu *et al.*^24^, the incidences of type I in mandibular first premolars were 72%, 50% and 65.2%, respectively. A 16.6% incidence of type II was found by Velmurugan and Sandhya^29^. However, Parekh *et al.*^30^ reported 5% for the incidence of type II in first premolars. The incidence of canal configuration type III was found to range from 3.62 to 5%, type IV was 1.5–25% and type V was 8–22.6%, according to other researchers.^24,28–31^

The incidences of canal configurations for mandibular second premolars according to Vertucci *et al.*^9^ were 97.5% type I and 2.5% type V. Similarly, Caliskan *et al.*^7^ reported 93.62% type I and 6.38% type V in a Turkish population. Sert and Bayirli^32^ showed that the incidence of the type I root canal configuration was more than the other types. Ok *et al.*^8^ found a 98.5% incidence of the type I root canal configuration moreover in their studies: type IV (0.6%) and type V (0.5%) were approximately the same. These results are consistent with our study.

## Conclusions

This is the first population-based Turkish Cypriot study that can serve as a guide to the root canals of premolar teeth for this ethnic group. These data can be compared to those of other populations and will facilitate diagnosis and treatment planning in Turkish Cypriot adults, which may be valuable for dental professionals who treat large numbers of Turkish Cypriot patients.

## Figures and Tables

**Table 1:** Classification of maxillary first premolars according to numbers of roots and canals per root

*Maxillary 1. Premolar*	*Single root*					*Two rooted*					*Three rooted*				
*Type*	*Right*		*Left*		*Total (%)*	*Right*		*Left*		*Total (%)*	*Right*		*Left*		*Total (%)*
	*F (%)*	*M (%)*	*F (%)*	*M (%)*		*F (%)*	*M (%)*	*F (%)*	*M (%)*		*F (%)*	*M (%)*	*F (%)*	*M (%)*	
*One canal at apex*															
Type I	5 (1.1)	5 (1.1)	5 (1.1)	5 (1.1)	20 (4.5)	—	—	—	—	—	—	—	—	—	—
Type II	27 (6.1)	10 (2.2)	26 (5.9)	8 (1.8)	71 (16.2)	—	—	—	—	—	—	—	—	—	—
Type III	—	1 (0.2)	—	1 (0.2)	2 (0.4)	—	—	—	—	—	—	—	—	—	—
															
*Two canals at apex*															
Type IV	31 (7)	38 (8.6)	31 (7)	40 (9.1)	140 (32)	53 (12.1)	46 (10.5)	48 (10.9)	49 (11.2)	196 (44.8)	—	—	—	—	—
Type V	2 (0.4)	—	—	1 (0.2)	3 (0.6)	—	—	—	—	—	—	—	—	—	—
Type VI	—	—	—	—	—	—	—	—	—	—	—	—	—	—	—
Three canals at apex	—	—	—	—	—	—	—	—	—	—	—	2 (0.4)	—	2 (0.4)	4 (0.9)

Abbreviations: F, female; M, male.

**Table 2:** Classification of maxillary second premolar according to numbers of roots and canals per root

*Maxillary 2. Premolar*	*Single root*					*Two rooted*					*Three rooted*				
*Type*	*Right*		*Left*		*Total*	*Right*		*Left*		*Total*	*Right*		*Left*		*Total*
	*F (%)*	*M (%)*	*F (%)*	*M (%)*		*F (%)*	*M (%)*	*F (%)*	*M (%)*		*F (%)*	*M (%)*	*F (%)*	*M (%)*	
*One canal at apex*															
Type I	62 (13.9)	51 (11.4)	53 (11.9)	54 (12.1)	220 (49.4)	—	—	—	—	—	—	—	—	—	—
Type II	36 (8)	24 (5.3)	38 (8.5)	26 (5.8)	124 (27.8)	—	—	—	—	—	—	—	—	—	—
Type III	—	5 (1.1)	—	3 (0.6)	8 (1.7)	—	—	—	—	—	—	—	—	—	—
															
*Two canals at apex*															
Type IV	10 (2.2)	17 (3.8)	11 (2.4)	9 (2)	47 (10.5)	9 (2)	10 (2.2)	9 (2)	6 (1.3)	34 (7.6)	—	—	—	—	—
Type V	2 (0.4)	2 (0.4)	5 (1.1)	—	9 (2)	—	—	—	—	—	—	—	—	—	—
Type VI	1 (0.2)	—	—	—	1 (0.2)	—	—	—	—	—	—	—	—	—	—
Three canals at apex	—	—	—	—	—	—	—	—	—	—	—	1 (0.2)	—	1 (0.2)	2 (0.4)

Abbreviations: F, female; M, male.

**Table 3:** Classification of mandibular first and second premolars according to numbers of roots and canals per root

*Type*	*Mandibular 1. Premolar*					*Mandibular 2. Premolar*				
	*Single rooted*					*Single rooted*				
	*Right*		*Left*		*Total*	*Right*		*Left*		*Total*
	*F (%)*	*M (%)*	*F (%)*	*M (%)*		*F (%)*	*M (%)*	*F (%)*	*M (%)*	
One canal at apex	—	—	—	—	—	—	—	—	—	—
Type I	131 (25.8)	99 (19.5)	126 (24.9)	97 (19.1)	453 (89.5)	113 (25.2)	100 (22.3)	119 (26.5)	101 (22.5)	433 (96.6)
Type II	—	—	2(0.3)	—	2 (0.3)	—	1 (0.2)	1 (0.2)	3 (0.6)	5 (1.1)
Type III	—	6 (1.1)	3 (0.5)	9 (1.7)	18 (3.5)	1 (0.2)	1 (0.2)	3 (0.6)	—	5 (1.1)
										
*Two canals at apex*										
Type IV	1 (0.1)	—	1 (0.1)	—	2 (0.3)	—	—	—	—	—
Type V	5 (0.9)	11 (2.1)	4 (0.7)	11 (2.1)	31 (6.1)	1 (0.2)	2 (0.4)	—	2 (0.4)	5 (1.1)
Type VI	—	—	—	—	—	—	—	—	—	—
Three canals at apex	—	—	—	—	—	—	—	—	—	—

Abbreviations: F, female; M, male.
